# Pathogenic and Saprophytic *Leptospira* Species in Water and Soils from Selected Urban Sites in Peninsular Malaysia

**DOI:** 10.1264/jsme2.ME12154

**Published:** 2013-01-30

**Authors:** Douadi Benacer, Pei Yee Woh, Siti Nursheena Mohd Zain, Fairuz Amran, Kwai Lin Thong

**Affiliations:** 1 Institute of Biological Sciences, Faculty of Science, University of Malaya, 50603 Kuala Lumpur, Malaysia; 2 Laboratory of Biomedical Science and Molecular Microbiology, Institute of Graduate Studies, University of Malaya, 50603 Kuala Lumpur, Malaysia; 3 Bacteriology Unit, Institute for Medical Research, 50588 Kuala Lumpur, Malaysia

**Keywords:** *Leptospira*, soil, water, MAT, PCR

## Abstract

*Leptospira* species were studied in water and soils from selected urban sites in Malaysia. A total of 151 water (*n*=121) and soil (*n*=30) samples were collected from 12 recreational lakes and wet markets. All samples were filtered and inoculated into semi-solid Ellinghausen and McCullough modified by Johnson and Harris (EMJH) media supplemented with additional 5-fluorouracil. The cultures were then incubated at 30°C and observed under a dark field microscope with intervals of 10 days. A PCR assay targeting the *rrs* gene was used to confirm the genus *Leptospira* among the isolates. Subsequently, the pathogenic status of the isolates was determined using primer sets G1/G2 and Sapro1/Sapro2, which target the *secY* and *rrs* genes, respectively. The isolates were identified at serogroup level using the microscopic agglutination test (MAT) while their genetic diversity was assessed by pulsed field gel electrophoresis (PFGE). Based on dark field microscopy, 23.1% (28/121) water and 23.3% (7/30) soil cultures were positive for *Leptospira* spp. Of the 35 positive cultures, only 8 were pure and confirmed as *Leptospira* genus by PCR assay. Two out of 8 isolates were confirmed as pathogenic, 5 were saprophytic and one was intermediate. These 8 isolates were negative for the 25 reference hyperimmune rabbit sera tested in the MAT. PFGE showed that all 8 of these environmental *Leptospira* spp. were genetically diverse. In conclusion, the presence of pathogenic *Leptospira* spp. in the urban Malaysian environment may indicate and highlight the importance of water screening, especially in recreational lakes, in order to minimize any chance of *Leptospira* infection.

Leptospirosis is an important global zoonotic disease and is caused by spirochetes from the genus of *Leptospira*. Two major species, including pathogenic strains of *Leptospira interrogans* and non-pathogenic or saprophytic strains of *Leptospira biflexa*, have been identified (19). The main reservoirs for pathogenic *Leptospira* are the rodents, including rats that may carry pathogenic serovars (37). Saprophytic species are naturally present in environmental water and soil and do not usually cause disease (22). Leptospirosis occurs when pathogenic species are transmitted into the bloodstream of humans via direct contact with contaminated urine of animal reservoirs or indirectly by contaminated water and soil (5). The symptoms of leptospirosis may vary from asymptomatic to fatal, according the phase of the infection. There are two phases of leptospirosis infection where they differ in signs and symptoms, that is anicteric and icteric leptospirosis (19). Leptospirosis is known to be an occupational disease, commonly occurring among farmers, veterinarian, abattoir workers and fishermen (28, 33); however, recreational activities such as water sports and travel to endemic countries have also been recognized as risk factors for this disease (35).

The high humidity and warm temperature of tropical and subtropical countries are ideal for *Leptospira* to survive for long periods in the environment. In Malaysia, several outbreak cases have been reported (18, 27). A recent outbreak of leptospirosis associated with a public recreational lake in Hutan Lipur Lubuk Yu, Maran, Malaysia has raised public concerns as it involved three fatalities (26).

Fletcher (8) reported the first fatal case of Malaysian human leptospirosis in 1925 due to *Leptospira* serogroup Icterohemorrhagiae. Subsequently, he identified other serovars, Icterohemorrhagiae, Hebdomadis and Pyrogenes. Between 1970 and 1986, Tan and co-workers reported nine different *Leptospira* serovars, including Pyrogenes, Autumnalis, Canicola, Hebdomadis, Icterohemorrhagiae, Pomona, Grippotyphosa, Celledoni and Sejroe from cases of leptospirosis in clinics and hospitals all over Malaysia (31, 32). In a recent study, the seroprevalence of leptospirosis among municipal workers in Kota Bharu, Kelantan, Malaysia showed that serovars Bataviae, Javanica and Patoc 1 were the 3 predominant *Leptospira* (29).

The isolation of zoonotic *Leptospira* was initiated by Gordon-Smith and co-workers who described 13 different *Leptospira* serogroups from rats (11). Apart from rats, other animal hosts included pigs, horses, dogs and cattle. The *Leptospira* serovars commonly involved in leptospirosis in animals are Pomona, Hebdomadis, Tarrasovi, Canicola and Hardjo (2).

Early research on the isolation of *Leptospira* from water and soil in Malaysia began in the 1970s (1, 3). To date, 29 pathogenic serovars have been identified in the Malaysian environmental water and wet soil (1). Ridzlan *et al.* (25) detected serovar Hebdomadis in water and soil samples collected from selected National Service Training Centres in Kelantan and Terengganu, Malaysia.

Culturing is used to isolate and maintain live cultures of different isolated *Leptospira* spp. The microscopic agglutination test (MAT) is widely used as the standard serology method to demonstrate the types of leptospiral serogroups based on the antibody-antigen reaction (19). A positive MAT is determined based on the agglutination of leptospiral cells with the reference hyperimmuned antisera tested. Rapid detection of *Leptospira* by polymerase chain reaction (PCR) has also been established (12, 21) as a useful tool in the detection of leptospiral DNA from human, animal and environmental samples (24, 34, 36). PFGE has proven to be a discriminative tool in the characterization of *Leptospira* strains (10). With its reliability, reproducibility and easy interpretation, PFGE is able to overcome some limitations of the culture and serological methods and is the method of choice for molecular characterization of *Leptospira* spp. (14).

The rapid urbanization of cities and improper garbage management system in urban areas probably created favorable conditions for animal carriers. This may pose a health risk for leptospirosis as infected animals and carriers might contaminate environmental waters and soils via their excreta and urine. The aim of this study was to detect and characterize *Leptospira* species in water and soils from selected urban sites. PCR assay was used for detection and differentiation between pathogenic and saprophytic species. Determination of different serogroups among the positive isolates was carried out by MAT, and the genetic relatedness among these Malaysian isolates was determined by PFGE.

## Materials and Methods

### Study sites

In the present study, the sites were chosen based on places frequented by the public, such as recreational parks, and drain effluents from high density residential homes. A total of 151 water (*n*=121) and soil (*n*=30) samples were collected from 12 selected sites in 3 different states (Kuala Lumpur, Selangor and Johor) in Peninsular Malaysia. One hundred and twenty-one water samples were collected from lakes, swamps and effluent drain waters, while 30 soil samples were collected from roadsides near housing areas, wet and night markets. Two types of soils, sand and loam, were recognized (Table 1). Samples were collected for 36 sessions during a period of 6 months (October 2011 to March 2012). The average temperatures in these 3 states ranged from 23°C to 34°C and rainfall averaged 240 cm year^−1^.

### Sample collection

Water and soil sample collections were carried out as described by Henry and Johnson (13), with some modifications. All the samples were collected in early morning. Approximately 100 mL water from four selected marked points of lakes and swamps were collected, poured into a 500 mL sterile glass bottle, and mixed thoroughly. Aliquots of 250 mL of well-mixed water samples were transported to the laboratory. From the street drain water, 100 mL surface water from each drain was collected and transferred into sterile glass bottles. Approximately 20 g topsoil (15 cm by 5 cm) was collected from wet and shaded areas. The soil was immediately placed in a sterile plastic bag. The temperatures of water and soil were recorded in the field and pH was recorded upon return to the laboratory. All the samples were transported to the laboratory and processed within 12 hours. The summary of water and soil sample collection is shown in Table 1.

### *Leptospira* isolation and dark field microscopy examination

Water samples (100 mL) were filtered through a sterile membrane filter with 0.45 μm pore size. One milliliter of filtered water was inoculated into modified semi-solid Ellinghausen and McCullough modified by Johnson and Harris (EMJH) medium. Soil samples (20 g) in a plastic bag were soaked in sterile phosphate-buffered saline (PBS) solution at approximately three times the volume of the samples. They were mixed by vigorously shaking and allowed to settle for 15 to 20 min. The suspension was pre-filtered through sterile filter paper (Whatman no.1) and then through a sterile 0.45 μm membrane filter. The filtered water (1 mL) was inoculated into EMJH culture media. All the inoculated media were incubated aerobically at 30°C for seven days.

Two types of culture media for *Leptospira* spp. were prepared: liquid and semi-solid EMJH media. The enrichment media contained 1.0% of rabbit serum and bovine serum albumin. Semi-solid EMJH media was prepared by adding 0.13% of Bacto agar to the liquid media. Both media were supplemented with 5-fluorouracil (Merck, Darmstadt, Germany) at a concentration of 400 μg mL^−1^ to minimize bacterial contamination. All the cultures were incubated at 30°C for 30 days and examined under a dark field microscope for the presence of *Leptospira* at intervals of 10 days. *Leptospira* were identified by their characteristic motility as well as morphology. *Leptospira* are white, very thin, long, and rotate rapidly on their longitudinal axis, moving backward and forward. The samples were considered negative if the bacteria were not present in the culture. The positive samples were subcultured into liquid medium and used for further analysis (25).

### PCR detection and confirmation of *Leptospira* spp

#### DNA template preparation

Genomic DNA was extracted from 7 days’ fresh culture using a Wizard Genomic DNA Purification Kit (Promega, Madison, WI, USA) following the manufacturer’s instructions. The quantity of DNA was measured by Biophotometer (Eppendorf, Germany).

#### PCR confirmation of *Leptospira* spp

To confirm the genus *Leptospira* and to determine the pathogenic status of the isolates, 3 published primers sets were used (12, 21, 23). In all PCRs, the reactions were performed in a final volume of 25 μL containing 1× PCR buffer, 1.5 mM MgCl_2_, 200 μM each of dNTPs, 0.3 μM of each primer, 1 U of *Taq* DNA polymerase (Intron Biotechnology, South Korea) and 100 ng template DNA. The PCR products were analyzed by electrophoresis through a 1% agarose gel (Promega).

Primers LA/LB were used to target the 16S rRNA gene as described by Merien *et al.* (21). The cycling conditions consisted of initial denaturation at 94°C for 3 min, 35 cycles each of 94°C for 1 min, 57°C for 1 min, 72°C for 2 min, and further extension at 72°C for 10 min. Primers G1/G2, which target the *secY* gene, were used to detect pathogenic *Leptospira* except for *Leptospira kirschneri* (12). The cycling conditions consisted of initial denaturation at 94°C for 10 min, 35 cycles each of 94°C for 1 min, 55°C for 1 min, 72°C for 1 min, and further extension at 72°C for 5 min. To detect saprophytic *Leptospira* among the isolates, Sapro1/Sapro2 primers were used (23). The cycling conditions consisted of initial denaturation at 94°C 10 min, 35 cycles each of 94°C for 1 min, 57°C for 1 min, 72°C for 1 min, and further extension at 72°C for 5 min.

#### DNA sequencing

Amplified DNA products from representative isolates were verified by DNA sequencing. The amplicons were purified using a DNA purification kit (Qiagen, Hilden, Germany) and submitted to a commercial facility for sequencing (First BASE, Pte., Singapore). The resulting DNA sequence data were compared with the GenBank database using the BLAST algorithm available on the web site (http://www.ncbi.nih.gov).

### Microscopic agglutination test

Serological identification of *Leptospira* isolates was performed using the microscopic agglutination test (MAT) as described by the World Health Organization (38). A set of 25 reference hyperimmune antisera representing the major *Leptospira* serovars in Malaysia was provided by the Institute for Medical Research (IMR), Malaysia. The antisera used in this study were: Patoc (Patoc I), Ballum (Mus 127), Sejroe (M84), Javanica (Veldrat Batavia 46), Tarassovi (Perepelicin), Bratislava (Jez Bratislava), Canicola (Hond Ultrecht IV), Hebdomadis (Hebdomadis), Pomona (Pomona), Hardjo (Hardjoprajitno), Australis (Ballico), Bataviae (Swart), Pyrogenes (Salinem), Icterohaemorrhagiae (RGA), Paidjan (Paidjan), Gurungi (Gurung), Djasiman (Djasiman), Bangkinang (Bangkinang I), Autumnalis (Akiyami A), Samaranga (Veldrat Sem 173), Proechimys (1161 U), Grippotyphosa (Mandemakers), Grippotyphosa (Moskva V), Cynopteri (3522 C) and Celledoni (Celledoni). The leptospiral isolates were cultured in liquid medium with an additional 1.0% rabbit serum to increase bacteria density. Agglutination of anti-leptospiral antibodies with live *Leptospira* was viewed under a dark field microscope. A positive MAT was scored when there was 50% agglutination, leaving 50% free cells as compared with the negative control (culture diluted 1:2 in phosphate-buffered saline only). Four known positive reference leptospiral cultures (Canicola, Pomona, Bataviae and Javanica) were included to test the viability of the antisera.

### Pulsed-field gel electrophoresis analysis

PFGE analysis was carried out according to a previous protocol (10) with minor modifications. DNA was digested with 10 U of restriction enzyme *Not* I (Promega) at 37°C. The restricted fragments were separated by PFGE in 0.5× TBE buffer, for 24 h at 14°C in a CHEF Mapper system (Bio-Rad, Hercules, CA, USA) using pulsed times of 2.2 to 35 s. *Xba*I-digested *Salmonella* Braenderup H9812 was used as the DNA size marker. PFGE data were analyzed using BioNumerics Version 6.0 (Applied Maths, Belgium) software. Clustering was based on the unweighted pair group average method (UPGMA) with position tolerance of 1.0.

## Results

Dark field microscopic examination showed that 35 (23.2%) of 151 samples (121 water, 30 soil) contained *Leptospira* isolates. The positive samples showed the typical morphology and characteristic motility of *Leptospira* genus; however, only 8 (22.9%) of these were pure, and the others (77.1%) were contaminated with a higher number of natural bacteria than the numbers of *Leptospira*. More *Leptospira* were found in the drain effluents compared to lake waters. From the 121 water samples, 28 (23.1%) positives were from drain effluents (*n*=21) and lake waters (*n*=7). Among the 30 soil samples, 7 (23.3%) showed positive isolates. The numbers of the positive leptospiral samples associated with different sampling sites are summarized in Table 1.

PCR for confirmation of *Leptospira* genus for the 8 positive pure cultures using LA/LB primers showed that all 8 isolates were *Leptospira* genus. Only 2 of these were pathogenic species as indicated by the presence of 240 bp amplicon. These 2 pathogenic isolates were isolated from drain effluents from Setapak (EW31) and Section 17 (EW77). DNA sequence analyses showed that EW31 and EW77 were closely related to *Leptospira alstonii* species (99% identity). One intermediate isolate (EW1) isolated from University Malaya Lake was 99% identical to *Leptospira wolffii*. Five isolates were confirmed as saprophytic using Sapro1/Sapro2 primers. These saprophytic isolates were isolated from drain effluents in Cheras (EW8), Pantai Dalam (EW107) and SS2 (EW49), and from lakes in Taman Jaya (EW42) and Taman Tasik Titiwangrsa (EW61). DNA sequencing analyses showed that EW8, EW42 and EW61 were 98% identical to *L. biflexa* and EW49 and EW107 were 99% identical to *Leptospira meyeri* species (GenBank accession nos. FJ812170, DQ991480 and HQ709385).

MAT analysis of the 8 confirmed *Leptospira* spp. using the 25 different hyperimmune antisera showed that none of the isolates was positive for the antisera used; however, a low titer toward serovar Patoc from *L. biflexa* species was observed in 3 saprophytic isolates (titer <1:40).

**Fig. 1:**
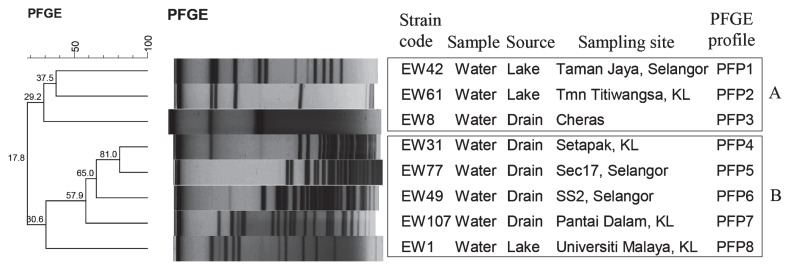
Dendrogram based on cluster analysis of the PFGE profiles of environmental *Leptospira* strains generated using Bionumerics Version 6.0 (Applied Maths, Belgium) software and unweighted pair group arithmetic means methods (UPGMA) 240×133 mm (96×96 DPI)

PFGE of *Not* I-digested chromosomal DNA subtyped the 8 isolates into 8 unique PFGE profiles (PFPs). The number of DNA fragments generated ranged from 2 to 23, with sizes ranging from 21 kb to 705 kb. Wide genetic diversity was found among the strains, as evidenced by F-values (F=0.2 to 0.8). The dendrogram showed 2 clusters, A and B (Fig. 1). Cluster A consisted of 3 isolates (EW42, EW61 and EW8), comprising 3 PFPs and cluster B consisted of 5 isolates (EW31, EW 77, EW49, EW107 and EW1), comprising 5 PFPs.

## Discussion

The majority of the leptospirosis cases reported in Malaysia was related to the exposure of humans to an environment contaminated by *Leptospira* spp. In 2000, an outbreak of leptospirosis occurred during the Eco-Challenge in Sabah, Malaysia. Eighty out of 189 competitors (42%) contracted leptospirosis. Twenty-nine people were hospitalized but there were no fatalities (27). The climatic conditions in this country, such as warm weather, heavy rainfall and high humidity, provide an appropriate niche for this organism to survive in the environment. In the past, leptospirosis was associated with occupational activities in rural areas, such as farming, rice harvesting, forestry and livestock farming (5, 19); however, leptospirosis has now become a public health problem in urban areas in many developing countries (15, 17). In Malaysia, there is a paucity of information about leptospirosis in the urban environment in terms of prevalence and the circulating species. Therefore, a study was carried out to investigate the prevalence and pathogenic status of this fastidious organism in the urban Malaysian environment. Samples were collected between October 2011 and March 2012 by sampling 12 randomly selected sites from 3 different states. The present study showed that the rate of positive samples (23%) collected from these sites was relatively high compared to another study by Ridzlan *et al.* (25), where they only found 10% (15/145) of positive water and soil samples in rural areas of Kelantan and Terengganu.

The presence of more *Leptospira* in drain effluent waters from night and wet markets compared to lake waters could be related to improper waste disposal. Rubbish not disposed of properly then becomes a food source for rats, cats, dogs and birds, which may be carriers of *Leptospira*. Several studies have reported that wild and domestic animals are maintenance hosts of *Leptospira* in Malaysia (2, 16). Three of five selected recreation lakes in Taman Jaya, Taman Tasik Titiwangsa and the University of Malaya were positive for *Leptospira*. Water in these lakes were nearly stagnant or showed a slow flow. Thus, there is a possibility that the lake waters were contaminated by the urine of domestic animals in the vicinity. The University of Malaya Lake is basically a man-made reservoir to meet the recreational and sporting needs of the students. The rubbish and garbage generated by the cafeteria near this lake provide an ample food source for rodents and cats that may carry this organism. In the recent outbreak in the Recreational Park in Maran, three fatal cases were reported. Water and soil samples from the outbreak site were found to be positive for *Leptospira* (26). The authorities suspected that water and soil were contaminated with urine of infected rats, as evidenced by the presence of dead rats and rat droppings near the food stalls in that area (26).

*Leptospira* is known to be sensitive to dryness; therefore, the soil samples had lower positivity because of their low water retention capacity and the sandy soil was relatively dry. Similarly, Khairani *et al.* (16) showed that serovar Hardjo has a higher survival rate in moist soils and at pH 6.9–pH 7.4. In Malaysia, the rainy season is between October and March. Several studies have reported the association between rainfall and the incidence of leptospirosis cases (5, 19). This may explain the high number of positive samples in our study. Between December 2006 and January 2007, an outbreak of leptospirosis involving 20 cases with 2 deaths occurred in Johor, Malaysia following a flood episode (20). Besides the rainfall factor, several other factors can affect the isolation of *Leptospira* from water and soil, such as pH, temperature, characteristics of water and soil and the presence of animals that are considered as reservoirs of *Leptospira* (25).

Among the 35 positive samples seen under the dark field microscope, only 8 (22.8%) were pure. The high contamination rate (77.2%) that we observed in the cultures made the isolation steps very difficult. Fast-growing contaminating microorganisms displaced slow-growing *Leptospira* in the enrichment medium such that this fastidious organism failed to establish itself in the medium (16). In this study, a pre-filter technique was applied using Whatman filter paper before filtration through a 0.45 μm syringe filter. This method helped to minimize bacterial contamination. In addition, the concentration of 5-fluorouracil selective antibiotics was gradually increased to inhibit the growth of other bacteria in culture media; however, this extra precautionary step did not remove all the microbial contamination. A recent study reported that the use of a combination of 5 selective antibiotics may help to prevent the growth of 16 microorganisms that are considered as possible contaminants during the isolation of *Leptospira* (7).

In the present study, specific PCR was able to confirm all 8 pure positive samples using *Leptospira* genus primers that target the *rrs* gene. Similarly, the two sets of primers that target the *secY* and *rrs* genes were useful and could differentiate the pathogenic and saprophytic isolates. The number of saprophytic isolates (*n*=5) was slightly higher than the number of pathogenic isolates (*n*=2). Pathogenic species of *Leptopsira* are probably less adapted to the environmental conditions than saprophytic species (4). The ability and usefulness of the PCR in this study proved its value in the detection and differentiation of *Leptospira* spp. In many studies, PCR is used for the early detection of *Leptospira* spp in clinical (9), animal (6) and environment (23) samples as it is specific and rapid. MAT is a serological test that is widely used to identify serogroups of *Leptospira*. In Malaysia, MAT with titer of >1:400 is considered positive. Although 25 different hyperimmune sera were used in this study, the serogroup of the 8 confirmed *Leptospira* isolates could not be identified. Additional sets of hyperimmune sera are required to further identify the serogroup of the isolates. Besides the requirement of a large number of antisera, MAT is tedious and time consuming (19). Due to these limitations, new molecular tools such as PFGE have been developed. In this study, PFGE analysis showed that the *Leptospira* isolates had distinct profiles. Two pathogenic isolates, EW31 and EW77, which were isolated from effluent drain waters, had close genetic relatedness (81% identity). According to Galloway and Levett (10), *Leptospira* isolates that shared ≥75% identity or with ≤3 band differences could be from the same species. This result concurred with the sequencing results, which showed that these 2 isolates had high identity (99%) to *Leptospira alstonii* species. Both isolates were from effluent drain water near wet markets where many rats were observed. Hence, we postulated that these 2 isolates were probably from the same origin. In previous studies, pathogenic *Leptospira* species isolated from water and soil belonged to *L. interrogans*, *Leptospira borgpetersenii* and *Leptospira kmetyi* (1, 25, 30). This is the first study to isolate *L. alstonii* and *L. wolffii* from water and soil in Malaysia.

In conclusion, this study has proven the presence of genetically diverse *Leptospira* in the Malaysian urban environment. Detection of pathogenic *Leptospira* in water samples especially in recreational lakes, may pose a health risk, especially to those who come into contact with contaminated water during sports activities. Necessary precautions should be taken by the authorities to monitor water bodies and to alert the public of contaminated water bodies in view of this. The presence of these pathogenic strains in effluent drains is also a concern for better control of the host reservoir population and appropriate garbage management.

## Figures and Tables

**Table 1:** Summary of water and soil sample collections and positive results

Sampling sites	Coordinates	Water				Soil			
	
		No. of samples	Average temperature	Average pH	No. of positive samples	No. of samples	Average temperature	Average pH	No. of positive samples
Kuala Lumpur:									
*Drain effluent water*									
1. Cheras	3°11′53″N 101°40′27″E	10	28°C	7.27	4	—	—	—	—
2. Pantai Dalam	3°6′49″N 101°39′45″E	10	28°C	6.47	1	10	28°C	6.54	3
3. Setapak	3°11′32″N 101°43′1″E	11	28°C	7.49	5	—	—	—	—
*Lake water*									
4. Taman Tasik Titiwangsa	3°10′42″N 101°42′25″E	10	30°C	7.55	1	—	—	—	—
5. Taman Setapak Jaya	3°11′27″N 101°43′41″E	10	29°C	7.01	0	—	—	—	—
6. Taman Tasik Perdana	3°8′30″N 101°41′4″E	10	30°C	7.54	0	—	—	—	—
7. UM Lake	3°7′9″N 101°39′26″E	10	29°C	7.76	1	—	—	—	—
Selangor:									
*Drain effluent water*									
1. SS2	3°7′6″N 101°37′17″E	10	28°C	7.57	6	10	28°C	6.56	3
2. Section 17	3°7′10″N 101°38′14″E	10	28°C	7.45	3	—	—	—	—
*Lake water*									
3. Taman Jaya	3°6′18″N 101°38′54″E	10	29°C	7.91	5	—	—	—	—
4. Taman Paramount	3°6′10″N 101°37′30″E	10	30°C	5.77	0	—	—	—	—
Johor:									
*Swamp water*									
1. Gemas Baru	2°34′46″N 102°36′43″E	10	28°C	6.63	2	10	28°C	6.38	1

Total	—	121	—	—	28	30	—	—	7

UM: University of Malaya
